# The Impact of Microplastic Particles on Population Dynamics of Predator and Prey: Implication of the Lotka-Volterra Model

**DOI:** 10.1038/s41598-020-61414-3

**Published:** 2020-03-11

**Authors:** Qi Huang, Yuyang Lin, Qiyin Zhong, Fei Ma, Yixin Zhang

**Affiliations:** 1 Department of Mathematical Sciences, Xi’an Jiaotong-Liverpool University, Suzhou, China; 2 Department of Landscape Architecture, Gold Mantis School of Architecture, Soochow University, Suzhou, China

**Keywords:** Ecological modelling, Population dynamics, Environmental impact

## Abstract

Microplastic particles are widely distributed in a variety of ecosystems and can be transferred to predators along a food chain after being ingested by prey. However, how microplastic particles affect prey and predator populations is not fully understood. In this study, using the Lotka-Volterra model, we theoretically investigated predator-prey population dynamics in terms of toxicological response intensity (strength to population growth rate) to microplastic particles, and examined the negative effects on prey feeding ability and predator performance due to microplastic particles. Results of numerical simulations indicate the critical properties of the predator-prey system in response to microplastic particles: (i) predators are more vulnerable than prey under exposure to microplastic particles; (ii) the effect of microplastic particles on prey and predator population growths can be negligible when toxicological response intensities of prey and predator are small; (iii) this system is prey dependent for predator functional response, whose stability highly relies on the density of prey; (iv) the reduced feeding capacity of prey and predator induced by microplastic particles does not significantly affect the population dynamics of the predator-prey system. Moreover, our analysis suggests that dynamic Lotka-Volterra models can play a vital role in predicting ecological impacts of microplastic particles on predator-prey population dynamics.

## Introduction

Microplastic particles (<5 mm in size) are widely distributed in all land and water ecosystems^1–3^. Both marine and freshwater microplastic particles can impact ecosystem health^4–6^. In particular, population dynamics of organisms can be altered by microplastic particles through trophic interactions^7,8^, such as bioaccumulation. Microplastic particles can be ingested by a variety of aquatic organisms, and will be transferred to predators along the food chain, thereby raising environmental toxin levels^9,10^ and causing damage to the physiological functions of organisms under a high concentration of microplastic particles^11^. In addition to the physical harm, toxicity can also arise from reduced feeding capability and abnormal behavior^9,12^. Moreover, aquatic organisms exposed to microplastic particles may experience reduced growth, as well as low reproduction and survival rates^13,14^. Although microplastic particles clearly have profound impacts on the population dynamics of organisms, little is known about the impacts of microplastic particles on predator-prey interaction and their population dynamics^15^. This study therefore examines how microplastic particles influence predator and prey population dynamics through the Lotka-Volterra predator-prey model.

The Lotka-Volterra model was initially proposed in the theory of autocatalytic chemical reactions^16^ and has been modified over the years by numerous researchers to make it more suitable for their particular application. For instance, the predator-prey version of the model is an extension of the logistic equation used to describe predator-prey population change^17^ and this study modifies the model to analyze the effects of toxicant accumulation and exposure to a toxicant on a system level^18^. Since microplastic particles do not easily biodegrade and can remain intact for centuries, they are similar to other persistent organic pollutants^19^. Thus, we consider the toxicological effect of microplastic particles to be analogous to this toxicant model. The study of a toxicant model of microplastic particles may provide insights for solving other plastic problems and persistent organic pollutants, as it concerns the environment and ecosystem^19^.

The objective of this study is to use a modified Lotka-Volterra model that combines the original Lotka-Volterra model^16^ with a single-species model under toxicant influence of microplastic particles^18^ to investigate how microplastic particles affect population dynamics of predator and prey populations in aquatic ecosystems. We discuss a simple system with only one predator and one prey. Furthermore, toxicological response intensity is a parameter about the growth rate of predator and prey in the reflection of microplatic particles, indicating the sensitivity to microplastic particles and the change of population dynamics due to toxicity of microplastic particles. Specifically, we test four hypotheses: (1) In the long run, microplastic particles will cause predator extinction eventually due to bioaccumulation in this system; (2) Population growth in the predator-prey system will not change much in the case of low response intensity (take value smaller than 1.0); (3) Coexistence of predator and prey depends on the abundance of prey; (4) The feeding capacity of prey and predator will greatly affect the population dynamics of this system. To observe the qualitative behaviour of the system, we plot the phase portrait of predator and prey. In addition, we use the time series graph to display the change in the number of predator and prey as time progresses.

## Material and Methods

### The model

Predator-prey interactions have been investigated systenmatically by following the work of Lotka^20^. In this section, we first introduce the Lotka-Volterra model and single-species model with toxicity factors. We then combine these two models to obtain a new modified model. The Lotka-Volterra predator-prey model is depicted by^16^:

$$\begin{array}{c}\frac{d{x}_{1}}{dt}={x}_{1}({r}_{10}-{r}_{11}{C}_{1}-{a}_{1}{x}_{2})\\ \frac{d{x}_{2}}{dt}={x}_{2}(-{r}_{20}-{r}_{21}{C}_{2}+{a}_{2}{x}_{1})\end{array}$$

In those two differential equations, ${r}_{10}{x}_{1}$, ${r}_{20}{x}_{2}$ are the intrinsic growth rate of prey and mortality rate of predator without toxicity, which is the same as conditions where prey reproduces exponentially in the system. We will show that this exponential growth can be limited by the bioaccumulation of microplastic particles. In addition, ${a}_{1}{x}_{1}{x}_{2}$ refers to the lost amount of prey eaten by a predator. The ${a}_{2}{x}_{1}{x}_{2}$ is the increasing number of predators due to the feeding of prey.

The single-species model with the effects of toxicants is established^18^, which provides views of adding microplastic particles toxicity into the predator-prey system:

2.1 $$\frac{dx}{dt}=x({r}_{0}-{r}_{1}{C}_{0}-nx)$$

2.2 $$\frac{d{C}_{0}}{dt}=S{C}_{E}-g{C}_{0}-m{C}_{0}$$

2.3 $$\frac{d{C}_{E}}{dt}=-\,{k}_{1}{C}_{E}x+{g}_{1}{C}_{0}x-h{C}_{E}+u(t)$$

In these formulas, *x*, C_0_ and *C*_*E*_ denote population size, amount of toxicity inside the species and amount of toxicity in the environment respectively. The prey is assumed to reproduce exponentially without toxicity in the original Lotka-Volterra model. *r*_0_ stands for this exponential growth in Eq. (2.1). In addition, to demonstrat the extent of toxicity influences, ${r}_{1}$ is utilized to denote the response intensity of toxicity. n is the restrictive factor of intraspecific competition here, but we will neglect effect of intraspecific competition and delete this term. As for Eq. (2.2), $S{C}_{E}$ denotes absorption rate of the toxicity. $g{C}_{0}$ and $m{C}_{0}$ represent the egestion rate and purification rate. In Eq. (2.3), ${g}_{1}{C}_{0}x$ and $u(t)$ denote the toxicity excreted by organism and the emission rate of toxicity to the environment respectively. ${k}_{1}{C}_{E}x$ and $h{C}_{E}$ represent the absorption rate of toxicity by organisms and the toxicity purification rate by the environment itself.

The significance of this model is that it provides terms ${C}_{0}$ and ${C}_{E}$ to present the toxicity effects on population dynamics. We can draw an analogy between toxicant and microplastic particles. Consequently, based on this toxic effect model, we add the influence of microplastic particles into the Lotka-Volterra, predator- prey model and produce a new model.

After combining a single-species model with the Lotka-Volterra model, we obtain the modified Lotka Volterra model:

2.4 $$\frac{d{x}_{1}}{dt}={x}_{1}[({r}_{10}-{d}_{1})-{r}_{11}{C}_{1}-({a}_{1}-{d}_{3}){x}_{2}]$$

2.5 $$\frac{d{x}_{2}}{dt}={x}_{2}[-{r}_{20}-{r}_{21}{C}_{2}+({a}_{2}-{d}_{2}){x}_{1}]$$

2.6 $$\frac{d{C}_{1}}{dt}={S}_{1}{C}_{E}-{g}_{1}$$

2.7 $$\frac{d{C}_{2}}{dt}={S}_{2}{C}_{E}+{\rm{k}}{C}_{1}-{g}_{2}$$

$${C}_{1},{C}_{2},{x}_{1},{x}_{2}\ge 0$$

This model demonstrates population dynamics of the predator-prey system with the influence of microplastic particles. We will use this modified model to simulate the predator-prey system in the later analysis. Three assumptions made in this model are:

1. The model neglects the influence of the intraspecific competition.
2. The effect of the microplastic is shown by its concentration.
3. The egestion rate ${g}_{1},\,{g}_{2}$ is independent of the microplastic concentration in the environment ${C}_{E}$, and the amount of microplastic concentration removed at each time step is independent of the total amount of microplastics in the organisms ${C}_{1}$ and ${C}_{2}$.

Equations (2.4) and (2.5) illustrate the toxicological effects of microplastics on the population of prey and predator. ${x}_{1}$, $\,{x}_{2}$ represent the population of prey and predator respectively. Microplastic particles have either negative or neutral effect on the reproduction and survival on aquatic organisms^13^. In particular, exposure to microplastic particles leads to behavioral abnormality including the interruption of feeding ability and the hypoactivity phenomenon^21,22^. Such detrimental effects can cause the inhibition of the intrinsic growth rate of prey. Thus, we use ${d}_{1}$ to denote the decline in the prey feeding ability. In addition, reduction of the predatory performance and efficiency is emphasized when exposed to microplastic particles^23^. Since ${a}_{2}{x}_{1}{x}_{2}$ measures the increasing number of predators due to the feeding of prey in the model, we apply ${d}_{2}$ to denote the adverse effect of reduced predatory performance, which can decrease the number of predators. Therefore, for the prey, the lost amount of prey eaten by the predator will decrease and is denoted by ${d}_{3}$. Furthermore, there is a potential for microplastic particles to cause other detrimental effects and damage to organisms, including inflammatory responses, oxidative damage, disruption of metabolism, immunity, and neurotransmission dysfunction^24–26^. Here, we utilize ${r}_{11}{C}_{1}$ and ${r}_{21}{C}_{2}$ to express these toxicological effects induced by microplastic particles, which describe the response intensity of prey and predator to the microplastic particles respectively. We will later simulate the predator-prey system according to the values of $\,{r}_{11}$ and ${r}_{21}$. Note that ${r}_{10}$, ${r}_{20}$, ${a}_{1}$ and ${a}_{2}$ are all positive parameters. In addition, we assume ${r}_{11}$ and ${d}_{1}$ have the same sign, while ${r}_{21}$, ${d}_{2}$ and ${d}_{3}$ have the same sign.

Equations (2.6) and (2.7) are related to the uptake, ingestion and egestion of microplastics. ${C}_{1}$, ${C}_{2}$ denoted average microplastic particles concentration distributed inside the body of prey and predator respectively. A review by Hidalgo-Ruiz *et al*.^27^ indicates that plastic particle selection occurs in the uptake of microplastics. Organisms may exert selectivity between particles, affecting the bioavailability of microplastics^28^. Physical factors of microplastics such as size, density, abundance and color can determine this bioavailability^9^. Therefore, we use ${S}_{1}$ and ${S}_{2}$ to present the effects of plastic particle selection of prey and predator respectively. Furthermore, ${S}_{1}{C}_{E}$ represents the microplastic particles absorption rate of prey and ${S}_{2}{C}_{E}$ represents the direct microplastic particles ingest rate by predator from the environment. $k{C}_{1}$ represents the accumulated toxicity of microplastic particles transferred from the prey, which is the effect of bioconcentration. As for the egestion of microplastics, Au *et al*.^29^ suggest that the microplastic egestion rate is not significantly different to the microplastic concentration and is related to the mechanism of the organism itself. Consequently, different from Eq. (2.2) and Eq. (2.3), toxicity effect of egestion is simplified in this modified model, and we use ${g}_{1},\,{g}_{2}$ to represent the microplastics egestion rate of prey and predator.

### Parameter estimation

With respect to parameter estimations, the concentration of microplastic particles in a freshwater environment (denoted by *C*_*E*_) is not a function of time, but will be affected by external factors such as rainfall, wind conditions as well as the local physical environment^30^. However, because this study is a theoretical analysis, we ignore the influence of external factors and just consider *C*_*E*_ as a constant in this model. Also, we use data from the literature and the value of concentration in surface water (30 particles/m^3^) in the simulation.F

Egestion times of prey (denoted by *g*_1_) and egestion times of predator (denoted by *g*_2_) are other two important parameters to estimate removal of microplastic particles. Au *et al*.^29^ proposed that microplastics egestion rate is related to the microplastics shapes, size and life-stage of the organism, but not significantly varied by the exposure concentrations. Egestion times remain unchanged at different exposure of microplastic particles concentration, and is similar to the natural food items. This may indicate that microplastics egestion rate is independent of its concentration. Therefore, in this study we assume the egestion rate ${g}_{1},\,{g}_{2}$ to be two constants.

If experimental data is available, the intrinsic rate of the increase of prey without microplastic particles (denoted by ${r}_{10}$) can be calculated. The computing method is to take the population after a time period minus the initial population and divide this result by the initial value^31^. Similarly, the increasing number of predators due to the feeding of prey (denoted by ${a}_{2}$) can be estimated by the same formula. Furthermore, the natural mortality rate of the predator (denoted by ${r}_{20}$) can be considered as the multiplicative inverse of ${a}_{2}$.

However, due to lack of experimental data, this study investigates a model population instead of a real population. We obtain proper parameters from an empirical study^32^ with the following values and initial conditions:

$$\{\begin{array}{l}{x}_{1}(0)=100\\ {x}_{2}(0)=10\\ {C}_{1}(0)=0\\ {C}_{2}(0)=0\\ {r}_{10}=4.1\\ {r}_{20}=4.0\\ {d}_{1}=0.1\\ {d}_{2}=0.002\\ {d}_{3}=0.002\\ {a}_{1}=0.052\\ {a}_{2}=0.052\\ {g}_{1}=1.2\\ {g}_{2}=1.3\\ {S}_{1}=0.042\\ {S}_{2}=0.039\\ k=2.0\end{array}$$

### Numerical simulation

**Figure 1:**
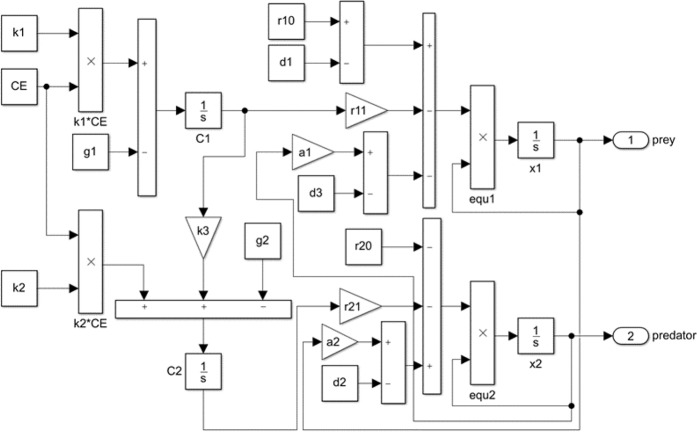
Simulink blocks’ diagram of Modified Lotka-Volterra equations. Simulink provides a simulation environment for editing the model. The integrator block 1/s outputs the number of predators and prey. All the simulations below use this diagram.

The model is implemented using MATLAB programming and its Simulink toolbox. Figure 1 shows the operation of Simulink. We can study the toxicity of microplastic particles by taking different values of response intensity of prey (*r*_11_) and predator (*r*_21_) to microplastic particles. Furthermore, influence of the decline in prey feeding ability and predatory performance is analyzed by taking different values of ${d}_{1}$, $\,{d}_{2}$ and ${d}_{3}$. For the convenience of discussion, we use letter $\Delta$ to denote proportion of ${r}_{11}$ and $\,{r}_{21}$ $(\Delta =\frac{{r}_{11}}{{r}_{21}})$.

## Results

We first investigate the toxicological effects of microplastic particles according to the value of response intensity ${r}_{11}$ and ${r}_{21}$. In this simulation, we consider ${d}_{1}$, ${d}_{2}$ and ${d}_{3}$ as constants and take values of 0.1, 0.002 and 0.002 respectively. We classify interactions to microplastic particles into four conditions to measure performance of population dynamics of predator and prey.

(Condition a): without the influence of microplastic particles $({C}_{1}=0,{C}_{2}=0,\,{r}_{11}=0,{r}_{21}=0)$.

(Condition b): predator and prey have same response strength to microplastic particles $(\Delta =\frac{{{\rm{r}}}_{11}}{{{\rm{r}}}_{21}}=1.0)$.

(Condition c): predator has much larger response strength than prey $(\Delta =\frac{{{\rm{r}}}_{11}}{{{\rm{r}}}_{21}}=0.1)$.

(Condition d): predator has much smaller response strength than prey $(\Delta =\frac{{{\rm{r}}}_{11}}{{{\rm{r}}}_{21}}=10.0)$.

We then consider the effects of the decline in the ability of prey feeding (related to ${d}_{1}$) and predatory performance (related to ${d}_{2}$ and ${d}_{3}$) to the population dynamics of the predator-prey system. We increase the value of ${d}_{1}$, ${d}_{2}$ and ${d}_{3}$ to 0.6, 0.012 and 0.12. We also refer to phase portrait and time series plot. The first graph is to explore the stability of the predator-prey system, and the second graph is the population of predator and prey.

### Four response intensities of prey and predator to toxicological effects induced by microplastic particle

**Figure 2:**
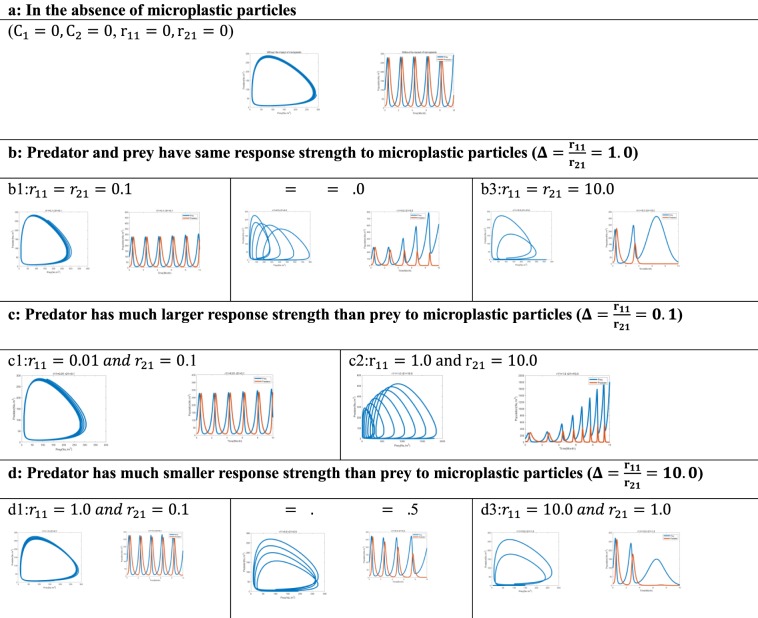
Phase portrait and short-term population dynamics of the predator-prey. Phase portrait depicts the trajectories of the predator-prey system, with the X axis showing population of prey (*x*_1_) and the Y axis the population of predator (*x*_2_). In time plot, X-axis measures time in months and Y axis measures the number of organisms (No./m^3^). Also, population of prey (*x*_1_) and population of predator (*x*_2_) are represented by blue full line and red full line respectively. Four conditions of different response strength in related to the population of prey and predator.

(Condition a): We first simulate the condition where no microplastic particles exist. Inside microplastic particles, concentrations of prey and predator and the response intensities are equal to zero (C_1_ = C_2_ = r_11_ = r_21_ = 0). Population of prey and predator can be stable in this case, and it fluctuates wildly with a high of 250 and a low of 20. In addition, the period is 2. By comparing these results with the simulations below, we can identify whether the predator-prey system will collapse due to microplastic particles.

(Condition b): Firstly, when effects of microplastic particles on prey and predator are both weak (r_11_ = r_21_ = 0.1). Figure 2b1 shows that the population of prey and predator fluctuates between 20 and 300, which does not change much compared with conditions in the absence of microplastic particles. Secondly, when effects of microplastic particles on prey and predator are both in the middle level, the number of prey is not the steady growth, but the growth of the fluctuation, especially the characteristic periodic increase (Fig. 2b2). However, the number of predators is generally decreasing with fluctuation and tends to become zero. Potential reason for this result is that: Microplastic particles reduce the number of predators, leading to the increase of prey. Although number of preys can be decreased by microplastic particles to a small extent, it is more than offset by less predators and lower predation rate. Therefore, the population of prey is trending upward, while the population of predators is trending downward. Conclusively, in this case, the predator tends to become extinct in the long run. Thirdly, when the effects of microplastic particles on prey and predator are very large. Note that after t = 3.4, the number of predators drops rapidly and becomes extinct (Fig. 2.b3). Nevertheless, the number of prey rises sharply at t = 3.8 and after peaking at t = 6.6, it decreases dramatically to zero. Consequently, when the impact of microplastic particles is severe, it can cause the extinction of both predator and prey.

(Condition c): The population of predator and prey is stable under the different but little effect of microplastic particles, and shows a similar trend as conditions in the absence of microplastic particles (Fig. 2.c1). When the impact on prey is small while impact on predator is quite large, we can see from Fig. 2.c2 that the population of the predator fluctuates mildly while the peak of prey increases gradually. Although r_21_ = 10.0 can cause the extinction of predator in the previous simulation, the increase of prey offset that influence in this case.

(Condition d): Firstly, the degree of fluctuation of prey and predator is not influenced greatly by microplastic particles under small response intensity of predator and prey (Fig. 2.d1). Secondly, the peak of each period of predator is decreasing, but the regularity of prey cannot be obtained by only using one figure (Fig. 2.d2). However, we predict that the number of prey may go up since the number of predator has already decreased. Thirdly, in the case when the effect on prey is severe while the effect on predator is relatively small. The number of predators still falls sharply at t = 3.0 and eventually levels off at zero (Fig. 2.d3). Consequently, even though the response intensity of microplastic particles on prey (*r*_11_) is much larger than that on predator (*r*_21_), the number of predators still decreases earlier than prey. This may be interpreted as a decrease of prey will limit the increase of predator.

### Negative effects of microplastic particles on prey feeding ability and predatory performance

**Figure 3:**
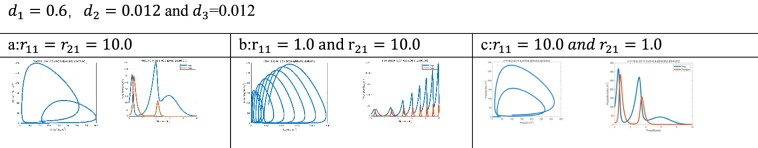
Three conditions of different response strength while value of ${d}_{1}$, ${d}_{2}$ and ${d}_{3}$ has increased to ${d}_{1}=0.6$, ${d}_{2}=0.012$ and *d*_3_ = 0.012. Phase portrait and short-term population dynamics of the predator-prey. Phase portrait depicts the trajectories of predator-prey system, with the X axis showing population of prey (*x*_1_) and the Y axis the population of predator (*x*_2_). In time plot, X-axis measures time in months and Y axis measures the number of organisms (No./m^3^). Also, population of prey (*x*_1_) and population of predator (*x*_2_) are represented by blue full line and red full line respectively.

When prey and predator have the same response intensity to toxicological effects of microplastic particles but worse prey feeding ability and weak predatory performance, as shown in Fig. 3.a–c, population dynamics of the prey-predator system show similar patterns and trends as Fig. 2. This suggests that the population dynamics of whole prey-predator system are not greatly influenced by the decline in prey feeding ability and predatory performance. However, subtle change can be observed from Fig. 3.a that prey achieves its extreme value earlier than Fig. 2.b3 at t = 6.5. Also, Fig. 3.c and 2.d3 show that the peak of prey falls from 150 to 50 under the same toxicological effects. Consequently, when the response intensities of prey and predator to microplastic toxicity are large, effects of microplastic particles on population dynamics of prey-predator system are not influenced by the decline in both prey feeding ability and predator performance, except a tiny decrease in prey number, which reveals that prey is relatively more susceptible.

### Comparisons

**Figure 4:**
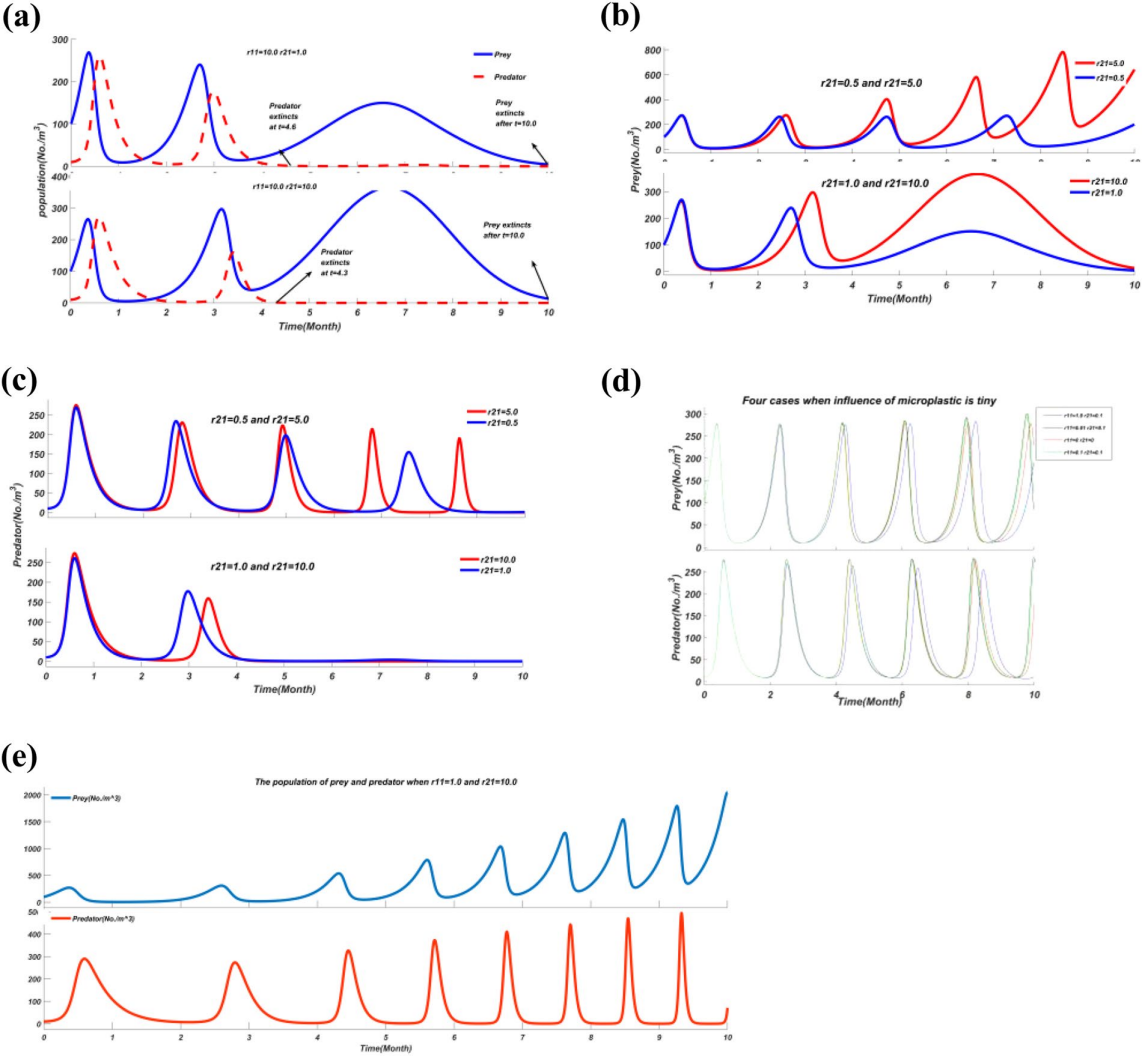
Five important comparisons of previews results. (**a**) Population dynamics of the predator-prey with response intensity of ${r}_{11}=10.0$, $\,{r}_{21}=1.0$ and when ${r}_{11}={r}_{21}=10.0$. Both prey and predator tend to extinguish in this graph. (**b**) Population dynamics of the prey with response intensity of prey (*r*_11_) same in each plot. (**c**) Population dynamics of the predator with response intensity of prey (*r*_11_) same in each plot. (**d**) Different short-term population dynamics of the prey and predator when response intensities of prey and predator are small (${r}_{11}=0\,{r}_{21}=0,\,{r}_{11}=0.1\,{r}_{21}=0.1,\,{r}_{11}=1.0\,{r}_{21}=0.1$ and ${r}_{11}=0.1\,{r}_{21}=0.1$). (**e**) Population dynamic of prey and predator with response intensity of prey equal to 1.0 $({r}_{11}=1.0)$ and response intensity of predator equal to 10.0 $({r}_{21}=10.0)$. For prey, the peak values of each period are 272.3, 310.5, 537.5, 788.8, 1040, 1291, 1544 and 1795. For predator, the peak values of each period are 289.5, 273.2, 326.5, 372.7, 402.7, 442.5, 469.7 and 493.6.

Firstly, under the circumstance where both prey and predator die out, the predator is extinguished earlier than the prey (Fig. 4.a). When the response intensity of prey $${r}_{11}$$ is tiny (Fig. 4.b), the population dynamic of prey shares the same trend, thus ($${a}_{2}-{d}_{2}){x}_{1}{x}_{2}$$ (increasing number of predators due to the feeding of prey) is not the reason for extinction of the predator. It can be seen from Fig. 4.c that population dynamics of predators fluctuate, which suggests that the growth rate of predators $$\frac{d{x}_{2}}{dt}$$ is not affected by microplastic particles. Consequently, by analyzing Eq. (2.5): $$\frac{d{x}_{2}}{dt}=-\,{r}_{20}{x}_{2}-{r}_{21}{C}_{2}{x}_{2}+({a}_{2}-{d}_{2}){x}_{1}{x}_{2}$$, it is found that the average microplastic particles concentration distributed inside a predator (*C*_2_) is the main factor to affect the number of predators. Secondly, when the response intensity of prey and predator ($${r}_{11}$$ and $${r}_{21}$$) is tiny, regardless of the proportion $$\Delta $$ $$(\Delta =\frac{{{\rm{r}}}_{11}}{{{\rm{r}}}_{21}})$$, curves of prey and predator almost coincide in the case without microplastic particles (Fig. 4.d). Thus, the variation among the population period of prey and predator due to weak effects of microplastic particles is slight. Thirdly, Fig. 4e depicts that the increasing rate of prey is faster than predators. Moreover, from Eq. (2.5) $$(\frac{d{x}_{2}}{dt}={x}_{2}[\,-\,{r}_{20}-{r}_{21}{C}_{2}+({a}_{2}-{d}_{2}){x}_{1}])$$, we can find $$\frac{d{x}_{2}}{dt} > 0$$ happens only when $$({a}_{2}-{d}_{2}){x}_{1}{x}_{2} > 0$$. This means that only when there is a relatively large population of prey (*x*_1_), can we find an increase of predators. Thus, the density of prey depends on the coexistence of predator and prey in this system.

## Discussion

Results reveal several ecologically important patterns: (1) Predators are more sensitive to the impact of microplastic particles; (2) When response intensities of predator and prey are weak, population dynamics of the predator-prey system are almost the same as in natural conditions; (3) This modified Lotka-Volterra predator-prey model is more prey dependent; (4) Decline of prey feeding ability and predatory performance does not have a great effect on the population dynamics of the predator-prey system. The following section explores the importance of these findings, and discusses the implications for the ecological coexistence of the predator-prey system.

### Predators are more vulnerable than prey under the influence of microplastic particles

The first hypothesis, that predators are likely to extinguish in the long run due to microplastic particles, is accepted to some extent. Our results suggest that predators are more sensitive to the effects of microplastic particles and that they always die out earlier than prey. This concurs with results from previous studies, which indicate that bioaccumulation of microplastic particles may be responsible for these phenomena. Mattsson *et al*. and Cedervall *et al*.^33,34^ confirmed that nano-sized microplastic particles can be transported to top-consumers, having adverse effects on their metabolism and behavior. Other studies also show that after being transported through food webs, microplastic particles and associated contaminants will accumulate and magnify into higher order predators^35,36^, which suggests that predators are more susceptible to the toxicological effects of microplastic particles^33,34,37^. This is significantly correlated with differential Eq. (2.6) $(\frac{d{C}_{1}}{dt}={S}_{1}{C}_{E}-{g}_{1})$ and Eq. (2.7) $(\frac{d{C}_{2}}{dt}={S}_{2}{C}_{E}+k{C}_{1}-{g}_{2})$ in our model, which explains that the concentration of microplastic particles in predators is higher than prey due to the term *kC*_1_ (bioaccumulation effect). To sum up, we conclude that bioaccumulation of microplastic particles (*k*) is the reason why predators are more vulnerable.

As one of the top predators in the ecological system, humans can be negatively affected by bioaccumulation of microplastic particles. The uptake of plastic particles by humans can occur through the consumption of terrestrial and aquatic food products, drinking water and inhalation^38,39^. However, it is also important to note that the modified Lotka-Volterra model used in this study focuses on the short-term performance of this system, and this study suggests that the system will collapse due to the continuous accumulation of microplastic particles.

### When response strength ${{\boldsymbol{r}}}_{{\bf{11}}}{\boldsymbol{,}}{{\boldsymbol{r}}}_{{\bf{21}}}$ are small, the influences of microplastic particles on both prey and predator can be neglected

Findings from our study support the second hypothesis that population dynamics of predator and prey will not change much when taking small values of response intensity to microplastic particles. Results show that there are slight variations among the population period of prey and predator due to weak effects of microplastic particles. In a short period, if the effect of microplastic particles is small enough, the performance of population dynamics is almost the same as in the absence of microplastic particles. Meanwhile, the peak and trough in each period are generally the same. These results are in agreement with Lin^40^, who found that survival rates of organism under low concentrations of microplastic particles were almost the same as the survival rates in natural condition without microplastic particles. Therefore, we can deduce that when the response intensity of prey and predator (${r}_{11}$ and ${r}_{21}$) is tiny, the population dynamic of prey and predator can be stable, and it is not affected by the proportion $\Delta$ $(\Delta =\frac{{{\rm{r}}}_{11}}{{{\rm{r}}}_{21}})$. Consequently, we conclude that when response intensities ${{\rm{r}}}_{11},{{\rm{r}}}_{21}$ are very small, the influence of microplastic particles on population changes in prey and predator may be ignored.

### Density of prey is the key factor to ensure stability of the predator-prey system

The third hypothesis is supported by our results suggesting that prey dominates the stability of the predator-prey system. A crucial element that needs to be taken into consideration in this model is the functional response, which is the rate of prey consumed by a predator^41,42^. Previous theoretical work has shown that most predator-prey interaction is predator-dependent functional response^43^, and it emphasizes that both predator and prey affect the functional response of the system^44,45^. However, our model highlights a significant prey dependence, which means that the density of prey alone dominates the response. Specifically, our results show that when the prey becomes extinct, the predator will be extinguished as well. Meanwhile, if prey increases, regardless of the impact of microplastic particles on predators, the predator-prey system remains stable and the predator would not be extinguished. The reason for differences in the results may be because of the bioaccumulation of microplastic particles as previously discussed. Therefore, we infer that the density of prey can determine the coexistence of prey and predator in this system.

### The influence of declined prey feeding ability and predatory performance on prey-predator population dynamics is small

Contrary to the fourth hypothesis, our results show that a decline in feeding ability of prey and predator induced by microplastic particles does not significantly influence the population dynamics in this system. Microplastic ingestion is known to impede the feeding of organisms, which can result in the reduction of biomass and reduced growth^46,47^. However, our simulation results do not support this expectation and only a slight reduction in the population of prey was observed. An explanation for this discrepancy, we can consider it from the view of predator’s functional response, which suggests that the predation rate depends on the abundance of prey and predator other than the low feeding capacity^41,42^. This study indicates that the negative impacts of a declining feeding capacity induced by microplastic particles on the population dynamics of the predator-prey system are small.

## Conclusion

Impacts of microplastic particles on predator, prey and their interaction in an aquatic environment were investigated by using a modified Lotka-Volterra model. Four important response patterns of the dynamics of predator and prey to microplastic particles are found. First, the predator is more vulnerable than prey to the detrimental impact of microplastic particles. Second, the coexistence of a predator and prey population relies on the existence of prey, and density of prey is the key factor to ensure stability of a predator-prey system. Third, under a low response intensity (values smaller than 1.0), the impact of microplastic particles on a model predator-prey system is very weak. Fourth, the decline in feeding ability of prey and predator induced by microplastic particles does not significantly influence the population dynamics of the predator-prey system. There are two important implications from this study. (1) For the long-term research, bioaccumulation of microplastic particles is a crucial factor in plastic pollution, which needs further investigation of their impacts on multiple species. The effects of bioaccumulation differ in various food web systems, and might perform different functions to impact predator-prey populations. (2) This modified model provides an innovative approach to the prediction of population dynamics of the predator-prey system under toxicological effects of microplastic particles or similar persistent organic pollutants. Thus, in future, by further modifying this Lotka-Volterra model, we can study the influence of microplastic particles or other plastic pollutants on the population dynamics of multiple predator and prey species.
